# Optimization of news dissemination push mode by intelligent edge computing technology for deep learning

**DOI:** 10.1038/s41598-024-53859-7

**Published:** 2024-03-20

**Authors:** JiLe DeGe, Sina Sang

**Affiliations:** 1 School of Journalism and Communication, Renmin University of China, Beijing, 100872 China; 2 Faculty of Communiation and Documentation, University of Granada, 18010 Granada, Spain

**Keywords:** Personalized news recommendation, Reinforcement learning (RL), Deep Deterministic Policy Gradient (DDPG), Q-leaning area under curve, Intelligent edge technology, Computational science, Computer science

## Abstract

The Internet era is an era of information explosion. By 2022, the global Internet users have reached more than 4 billion, and the social media users have exceeded 3 billion. People face a lot of news content every day, and it is almost impossible to get interesting information by browsing all the news content. Under this background, personalized news recommendation technology has been widely used, but it still needs to be further optimized and improved. In order to better push the news content of interest to different readers, users' satisfaction with major news websites should be further improved. This study proposes a new recommendation algorithm based on deep learning and reinforcement learning. Firstly, the RL algorithm is introduced based on deep learning. Deep learning is excellent in processing large-scale data and complex pattern recognition, but it often faces the challenge of low sample efficiency when it comes to complex decision-making and sequential tasks. While reinforcement learning (RL) emphasizes learning optimization strategies through continuous trial and error through interactive learning with the environment. Compared with deep learning, RL is more suitable for scenes that need long-term decision-making and trial-and-error learning. By feeding back the reward signal of the action, the system can better adapt to the unknown environment and complex tasks, which makes up for the relative shortcomings of deep learning in these aspects. A scenario is applied to an action to solve the sequential decision problem in the news dissemination process. In order to enable the news recommendation system to consider the dynamic changes in users' interest in news content, the Deep Deterministic Policy Gradient algorithm is applied to the news recommendation scenario. Opposing learning complements and combines Deep Q-network with the strategic network. On the basis of fully summarizing and thinking, this paper puts forward the mode of intelligent news dissemination and push. The push process of news communication information based on edge computing technology is proposed. Finally, based on Area Under Curve a Q-Leaning Area Under Curve for RL models is proposed. This indicator can measure the strengths and weaknesses of RL models efficiently and facilitates comparing models and evaluating offline experiments. The results show that the DDPG algorithm improves the click-through rate by 2.586% compared with the conventional recommendation algorithm. It shows that the algorithm designed in this paper has more obvious advantages in accurate recommendation by users. This paper effectively improves the efficiency of news dissemination by optimizing the push mode of intelligent news dissemination. In addition, the paper also deeply studies the innovative application of intelligent edge technology in news communication, which brings new ideas and practices to promote the development of news communication methods. Optimizing the push mode of intelligent news dissemination not only improves the user experience, but also provides strong support for the application of intelligent edge technology in this field, which has important practical application prospects.

## Introduction

With the popularity of smart portable devices and algorithm push technologies, the research on personalized news recommendation algorithms has gradually increased in recent years. The application of personalized news recommendations is no longer rare, and most are continuously updated to improve the development space of the algorithm^1^. The newsroom based on Artificial Intelligence (AI) has five functions: cloud-intensive editing, intelligent review, intelligent poster, multi-mode search, and one-click function, which involves multiple links such as news editing, review, production, and search. The new newsroom enables AI to see, do and review, which greatly improves the efficiency of traditional news editing and comprehensively transforms to digital intelligence. However, the social problems brought about by personalized news recommendations are endless, and improving the algorithm will be of great significance to society's information security and quick information acquisition^2,3^. Under the influence of the "Internet+" model, intelligent edge computing technologies such as voice and text recognition, intelligent search, and accurate algorithm push play an important role in news communication. Intelligent edge computing technology reshapes the business form of news media. The penetration of this technology in the news communication industry has improved the efficiency of news production by leaps and bounds. The information explosion brought by big data has expanded traditional information channels unprecedentedly. News dissemination is more rapid, accurate, and personalized. The application trend of various intelligent edge computing technologies makes the traditional news communication industry face new possibilities and challenges.

The research on personalized news recommendation algorithms first started in 1995. Participants proposed a personalized service system at the American Association for AI. Subsequently, personalized news information services began to develop vigorously^4^. Many well-known news organizations are starting to personalize news recommendations for their clients. In the field of news recommendation, researchers are committed to discovering the news users like to browse and try to recommend the news that users are most likely to click to browse. This increases the user click rate, dwell time, and retention rate and obtains higher advertising profits^5,6^. The accuracy, coverage, diversity, and other indicators of the recommendation results are the basis for improving the user click rate, staying time, and retention rate^7^. Personalized news recommendations can allow users to obtain the most interesting content in the fastest time, increasing the comfort of use and stickiness of the platform^8^. The personalized push of information can be used in personalized news recommendations and Internet shopping, browsing news, checking the weather, and making restaurant reservations^9,10^. In the sales platform, researchers are committed to discovering the user's hobbies and hobbies for products and recommending the products that users are most likely to buy for users to browse to increase sales profits^11^. Recommendation algorithm is a kind of computer program or system applied to information filtering and personalized recommendation. Its main goal is to predict the items that users may like or be interested in, and provide them with personalized recommendation lists. This kind of algorithm uses the user's historical behavior, preferences or other relevant information, infers the user's possible preferences by analyzing these data, and generates recommendations according to the prediction results. Recommendation algorithms have been widely used in e-commerce, social media, music and video streaming media, providing users with more personalized and interesting experiences. Common recommendation algorithms include collaborative filtering, content filtering and model-based methods, which shows their respective advantages and applicability in different scenarios^12,13^. Collaborative Filtering (CF) was first applied to the field of recommendation algorithms to filter many spams. Then, the recommendation algorithm has become popular^14^. Since then, to improve the CF algorithm's recommendation accuracy, scholars have proposed a content-based recommendation algorithm. This algorithm completes the recommendation task by studying the browsing habits of users^15^. After that, a hybrid idea was proposed in the recommendation field. Researchers have mixed multiple algorithms to get better results^16^. Later, academia began to study model-based recommendation algorithms. Currently, this recommendation algorithm mainly uses statistical learning to model the data. Algorithms currently mainly include recommendation technology based on the Bayesian network, dimensionality reduction technology, recommendation algorithm based on clustering, and recommendation algorithm based on implicit semantics^17,18^. With the development of deep learning, recommendation systems based on neural network structures have begun to achieve good results^19^. Ayata et al. used Convolutional Neural Network (CNN) for a music recommendation system. After processing audio content information through CNN, the recommendation effect was better than the original music recommendation algorithm^20^. After that, Alharbi used CNN to process user-related behavioral information for modeling, which also had a gratifying recommendation effect^21^. Bobadilla et al proposed a method based on classification, which not only returned the score predictions, but also provided their reliability. Additional information (prediction reliability) can be used in many related fields of collaborative filtering, such as detecting tampering attacks, recommending explanations or navigation tools to show the dependence between users and items^22^. Caro-Martínez et al. put forward an example explanation method of recommendation system based on knowledge map, which had nothing to do with the model to take advantage of this knowledge demand. It only needed information about the interaction between users and projects. By properly transforming these knowledge maps into project-based and user-based structures, link prediction technology was applied to find the similarity between nodes and determine the explanatory items recommended by users^23^. Deepak proposed OntoInfoG++, which was a knowledge-centered infographic recommendation method, including merging metadata from multiple heterogeneous sources and crowdsourcing ontology to recommend infographics according to the topics that users were interested in. This method models interested user topics from query words, current user clicks and standard knowledge stores (such as BibSonomy, DBpedia, Wikidata, LOD Cloud and crowdsourcing ontology). Semantic alignment was achieved by using three different metrics, namely Horn index, EnAPMI metric and information entropy^24^. By calculating the semantic similarity between the rich topics of interest and the infographic labels, and arranging the recommended infographic in the order of increasing semantic similarity, the time sequence of meaningful infographic arrangement is generated to obtain the resulting infographic recommendation. The main objectives of this paper mainly included RL, recommendation system and information retrieval. The application of RL makes the recommendation algorithm more flexible to adapt to the changes of users' interests and improve the personalized recommendation effect of the system. In-depth research in the field of recommendation system is helpful for mining user behavior patterns, while the technology in the field of information retrieval provides support for processing and organizing a large amount of data more effectively. Previous studies have made some contributions to solving similar problems. Previous research on recommendation algorithms mainly focused on collaborative filtering, content filtering and other traditional methods, but the innovation of this study is to introduce Q-Leaning Area Under Curve (QAUC) model, combined with RL, and more comprehensively consider the user's historical behavior and system recommendation decision to improve the accuracy and adaptability of recommendation. This innovative fusion has injected new impetus into the field of recommendation algorithm, which is expected to further expand the research and application of personalized recommendation system. Table 1 below shows the types, advantages and disadvantages of recommended algorithms:

**Table 1:** Summary of recommended algorithm classification, advantages and disadvantages.

Classification name	Classification criteria	Advantages and disadvantages of classification	Application area
Collaborative filtering	On the basis of users or items	Advantages: simple and intuitive, suitable for sparse data; Disadvantages: cold start problem, data sparsity	E-commerce, social media
Content filtering	On the basis of item attributes	Advantages: solve the cold start problem and have strong personalization ability; Disadvantages: it is difficult to obtain information	News, books and music recommendation
The model-based method	Model using machine learning	Advantages: it is suitable for modeling complex relationships; Disadvantages: high requirements on data quality and quantity	Film and television, advertising recommendation

In Table 1 above, recommendation algorithms can be divided into different categories, such as collaborative filtering, content filtering and model-based methods. In the current research of recommendation system, the QAUC model combines RL algorithm, which is unique and innovative. By combining QAUC evaluation method, the model not only considers the historical behavior of users, but also optimizes the recommendation decision through RL, thus improving the recommendation accuracy and enhancing the adaptability of the system to the changes of users' interests. This paper introduces new ideas in the field of recommendation system, emphasizes the necessity of RL in personalized recommendation, and has significant innovation potential.

In the first section of this paper, the research background, research purpose and current research status are summarized and described, and then the architecture of the algorithm proposed in this paper is introduced. Finally, the performance of the algorithm is tested and evaluated. The significance of this paper is to propose an innovative OAUC model and integrate RL algorithm into recommendation system. By combining Q-learning and area under curve evaluation method, the model can not only consider the historical behavior of users, improve the accuracy of recommendation, but also have the ability to adapt to the changes of users' interests. This paper fills the gap in the field of recommendation system, emphasizes the necessity of RL in personalized recommendation, and provides new ideas and methods for promoting the development of recommendation algorithm, which has significant theoretical and practical significance. In this paper, click behavior data is used to optimize news recommendation strategy through DDPG RL algorithm to improve user click rate. In the experiment, QAUC curve is used to evaluate the performance of the model comprehensively, and the trade-off between accuracy and recall rate under different click probabilities is considered. The binary classification method is used to model the click behavior to ensure that the model accurately predicts whether the user clicks on the news. This paper method pays attention to details, and aims to explore innovative recommendation algorithms, making them more adaptable to changes in user interests and dynamic system updates.

## Materials and methods

### Recommender system architecture

**Figure 1:**
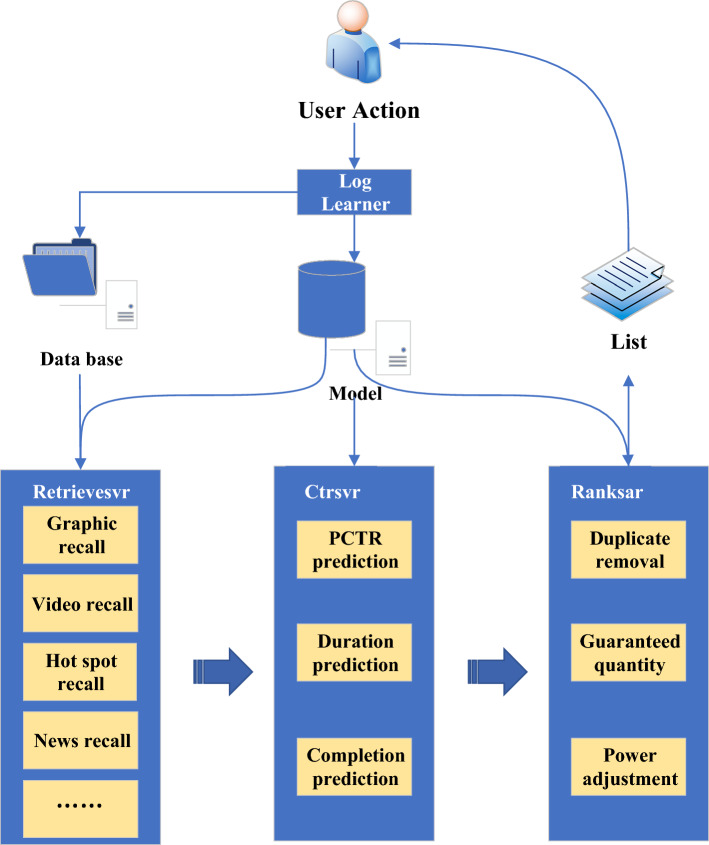
Architecture of a recommender system.

At present, the mainstream recommendation system architecture mainly includes four parts: the underlying basic data, the storage of data analysis, the recommendation calculation, and the business application^25^. In Fig. 1, Date-base is mainly used to store the underlying basic data and the profile and feature information obtained by analyzing the basic data and behavior logs. The recommendation calculation part mainly includes the recall and the ranking layer. The sorting layer can be further subdivided into a coarse and a fine sorting layer. The data obtained by means of hotspots, collaboration, and recall of portraits will first pass through the coarse sorting layer. After that, the fine ranking layer is further sorted by the click rate, duration, and completion degree prediction. Finally, the rule operations such as deduplication, quantity preservation, and weight adjustment are performed, and the recommendation result is obtained. Some models involved in the recommendation calculation are mainly obtained based on learning and training. Its offline training data can be extracted from the user's behavior log. The recommendation results may be applied to different businesses. A new recommendation list is generated and recommended to users, as shown in Fig. 1.

In Fig. 1 above, in the recommendation algorithm designed in this paper, there are four key parts, each of which plays a specific role. The underlying basic data is the basis of the recommendation algorithm, including the historical behavior data of users and the attribute information of items. These data are used as the input of the algorithm to analyze user preferences and behavior patterns. The quality and diversity of the underlying basic data are very important for the accuracy and personalization of the recommendation algorithm. In the process of data analysis and storage, the underlying basic data is stored after processing and analysis to support the subsequent recommendation calculation. This includes the mining of user behavior and the analysis of item attributes. Storing the processed data is helpful to improve the efficiency and response speed of the algorithm and provide the necessary input for recommendation calculation. Recommendation calculation is the core of the whole algorithm, and the stored data is used for model training, feature extraction and other operations to generate the final recommendation result. This section may include various recommendation algorithms, such as collaborative filtering and deep learning model, to ensure accurate and personalized recommendation. The final output of the recommendation algorithm is applied to practical business scenarios, such as recommending goods to users on the e-commerce platform and recommending songs on the music platform. Business application is the interface between the whole recommendation system and users, and the quality and practicability of recommendation results have a direct impact on user experience and business effect.

### RL

**Figure 2:**
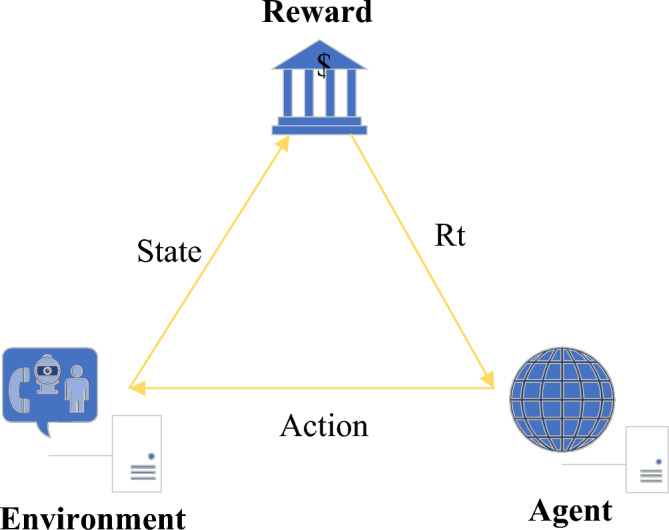
Basic structure of RL.

RL is a branch of Deep Learning (DL). The most prominent advantage of RL is the ability to learn from interactions^26^. In interacting with the environment, the agent continuously learns about the environment or completes certain tasks according to the reward or punishment obtained^27^. Usually, RL contains four elements: (A, S, R, and P). A represents all the actions of the agent; S represents the environmental states perceived by the intelligence; R represents the reward brought by the action, which is a value; P represents the strategy (or probability function). Its basic structure is shown in Fig. 2.

Q-Leaning is a common RL algorithm whose basic idea is Value-Based. Q(s,$\alpha$) means that the state at a certain moment is *s(s ∈ S)*. The policy gradient algorithm that the agent can get by selecting action an (a* ∈ A*) is different from the Q-Leaning algorithm. Gradient policy algorithms set a value function and use an algorithmic policy (such as a greedy algorithm, etc.) to select a certain action. RL can be divided into two categories: model-free and model-based. A model is a simulation of a real environment. The main difference between Model-free and Model-based is whether the environment model is known or needs to be calculated. Model-free is further divided into policy gradient-based and value-based. Model-based allows the agent to simulate actions and states according to the model and plan the action path in advance. However, there is an error between the model and the real environment. This error can cause an agent to perform well on the model but not in a formal setting. The implementation and parameter tuning of the Model-free algorithm is more friendly, and this type of algorithm is more popular and has more applications. This study mainly discusses algorithms such as Model-free.

### Deep RL

In this paper, RL algorithm is introduced to overcome the limitations of traditional recommendation algorithm in dealing with personalized recommendation. Compared with the traditional regression model, RL algorithm can better capture users' dynamic interests and behavior patterns through interactive learning with the environment. This introduction is based on the pursuit of further optimization of the recommendation system. By emphasizing the ability to learn and adapt to the individual needs of users, the effect and adaptability of the recommendation algorithm are improved. In machine learning research, DL has strong perception ability but lacks judgment and decision-making ability. RL has better decision-making ability and lacks perception. Therefore, the combination of DL and RL provides solutions and methods for more complex problems requiring perception and decision-making^28^. RL is characterized by interaction. The goal is to achieve the maximum cumulative return. Any calculation process can increase the reward value of the agent in the interaction process. In the news communication recommendation system, the use of deep RL affects the next decision of the system in the news recommendation system and applies a scenario to an action. The system determines which operations can produce the most returns through users' attempts. An action will affect the current immediate return and the next state. The subsequent return of the next state will be affected. Deep RL algorithms have been proposed for a long time, and researchers have also proposed many related algorithms. Deep Q-network (DQN) is a fusion of CNN and Q-Leaning. The advantage of this algorithm is that it can extract spatial information and learn appropriate control strategies from the original image data^29^. In the DQN algorithm, the calculation method of the objective function is shown in Eq. (1).

1 $${y}_{j}=\left\{\begin{array}{l}{R}_{j } \; is \;end=true\\ {R}_{j}+\gamma \,{\max}_ {\mathop \alpha \limits^{\prime}} Q\left(\varphi \left(\mathop {S_{j} } \limits{^{\prime}}\right), {\mathop {A_{j} }\limits}{^{\prime}}, w \right) \; is \; end=false\end{array}\right\}$$

In Eq. (1), the target Q value is calculated using the parameters of the currently trained Q network; *φ* and *γ* are the calculation parameters. However, practical applications prefer to update the parameters of the Q network based on ${y}_{j}$. ${A}_{j}$ represents the corresponding action of data element j. W represents the corresponding weight. DL and RL have very strong dependencies. In the iterative process, such a high correlation can easily cause the problem that the algorithm is not easy to converge. Meanwhile, the iterative process of DQN is shown in Eq. (2).

2 $$Q({S}_{t},{A}_{t})\leftarrow Q({S}_{t},{A}_{t})+\alpha \left[{R}_{t+1}+\gamma maxQ({S}_{t+1},\alpha )-Q({S}_{t},{A}_{t})\right]$$

There are two problems with such an iterative process. (1) The Q function puts the state ${S}_{t}$ and the action ${A}_{t}$ together for learning optimization, there is an action-independent deviation between different states. There are natural differences in the click-through rate of different recommended positions, and the relationship with the action is very small. (2) Max easily leads to the problem of overestimation, which makes the Q value of the model deviate greatly from the real value. The Q function is disassembled, as shown in Eq. (3).

3 $$Q\left({S}_{t},{A}_{t},\alpha ,\beta \right)=V({S}_{t},\alpha )+A({A}_{t},\beta )$$

**Figure 3:**
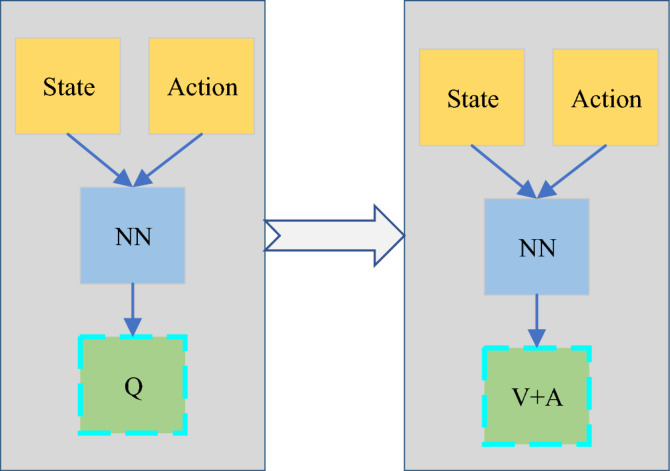
Evolution of DQN to Dueling DQN.

$\alpha \; {\text{and}} \; \beta$ are optimized states and actions. The Q function is a superposition of the state function V and the state-based action function A. Scholars proposed the Dueling DQN, whose structure is shown in Fig. 3. The main improvement of the neural network is that the Q value is obtained by adding the state and the action value A.

In Fig. 3 above, Dueling DQN (Deep Q-Network) structure is an innovative network architecture based on deep reinforcement learning (RL). Its core idea is to decompose the action value function into two parts: state value function and dominance function to estimate the value of each action more accurately. This structure consists of two independents fully connected neural networks, which are used to estimate the state value and dominance value respectively, and then calculate the final action value through a specific combination method. This decomposition enables Dueling DQN to learn the basic value of the state and the advantages of each action relative to the basic value more effectively, thus improving the accuracy of estimating the action value in complex environment. Decoupling the computation of the target Q value and the selection of actions is used to eliminate the overestimation problem, as shown in Eq. (4).

4 $$Q({S}_{t},{A}_{t})\leftarrow Q({S}_{t},{A}_{t})+\alpha \left[{R}_{t+1}+\gamma max \mathop Q\limits^{\prime} ({S}_{t+1},\alpha )-Q({S}_{t},{A}_{t})\right]$$

**Figure 4:**
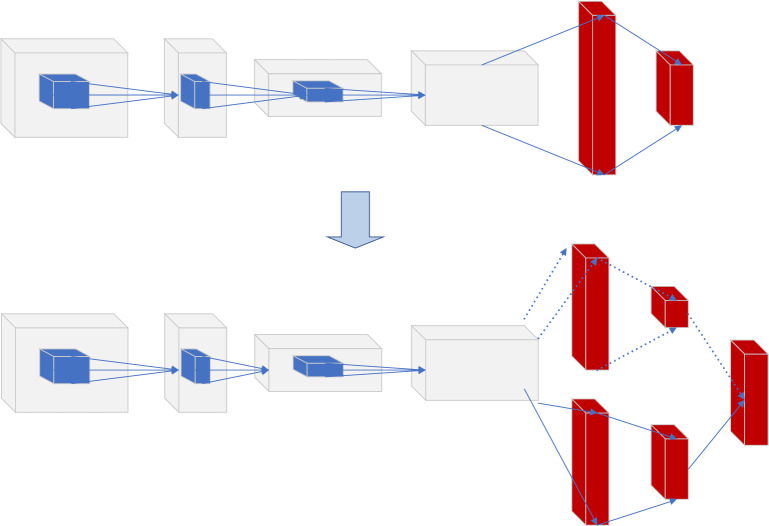
Evolution of DQN to dueling DQN.

The schematic structure of Double DQN is shown in Fig. 4. Compared with the DQN algorithm, Double DQN is divided into two parts: the main network and the target network. The main network is responsible for selecting actions; the target network performs Q-value fitting.

### The role of intelligent edge computing technology in news communication mode

The emergence of intelligent edge computing technology has constructed a new communication ecological model. The early digital transformation requires human resources to transform the data of the physical world into the digital world. As time goes by, digital transformation has gradually shifted from relying on human resources to AI^30^. Therefore, a new intelligent edge computing mode is proposed to enable each edge device of the Internet of Things (IoT) to have intelligent data collection, analysis and calculation, communication, and other capabilities. Edge architecture places data or applications at the edge of the network. Users can access data or applications at the network's edge, reducing bandwidth load and resource waste. Edge mode can be used for any work^31^. For example, retailers can use edge infrastructure to process purchase transactions in local stores. This operation can prevent purchase problems caused by transmission delays. Enterprises can store in nearby data centers for faster recovery next time. Intelligent edge computing can simultaneously use the capabilities of cloud computing to configure, deploy and manage edge devices on a large scale. It can allocate device types and scenarios intelligently to achieve more intelligent flow between cloud and edge. Intelligent edge computing applications in small scenarios have low requirements for latency, Quality of Service (QoS), etc. However, in large-scale scenario applications, the requirements for real-time data throughput, reliability, connection distribution, etc., are high and need to be customized. Based on intelligent edge computing technology, enterprises can use edge devices' computing and processing capabilities to directly handle most nearby IoT tasks. This can reduce the data center's workload and more timely and accurately respond to the different states of edge devices, making edge devices truly intelligent.

**Figure 5:**
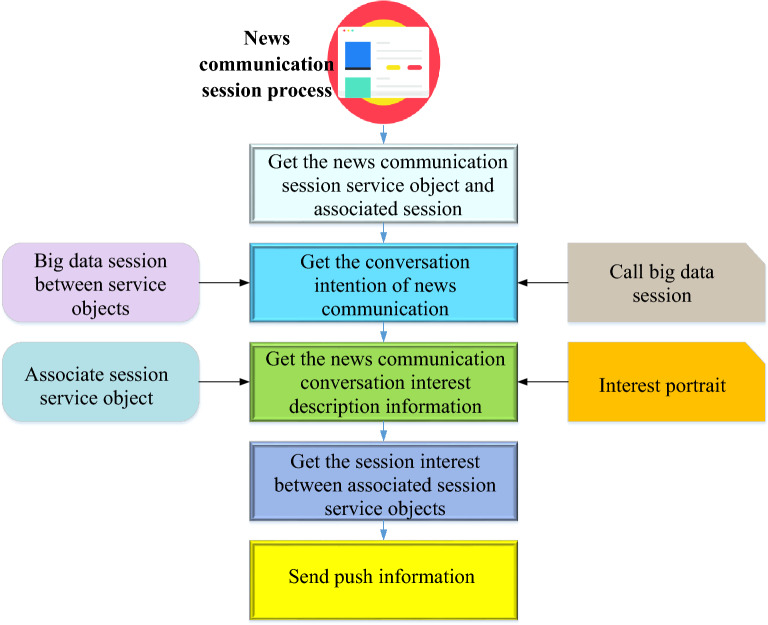
News dissemination push process based on intelligent edge computing technology.

The traditional mode of news communication is content production for a wide audience. Users can only find the content of interest in massive information. The emergence of intelligent edge computing technology has changed this situation. Data shows that among all business links of China’s news work, the application penetration of intelligent edge computing technology is most concentrated in public opinion monitoring, clue collection, accurate content dissemination, user portraits, etc. Intelligent edge computing technology Big data processing technology enables the audience's information needs to be amplified and accurately calculated. This enables the accurate dissemination of massive amounts of information, meets the audience's requirements in the digital age and improves the timeliness of news releases, personalized accuracy, and news production efficiency^32,33^. Most of the application operations of intelligent terminals provide the message push function, such as hot news recommendations of news clients, chat message reminders of interactive chat tools, e-commerce product promotion information, notification and approval process of enterprise applications, etc. Information push improves product activity, functional module utilization, user stickiness, and user retention. The news dissemination push algorithm is the key channel in the operation of news dissemination applications. The news dissemination push process based on intelligent edge computing technology is shown in Fig. 5.

In Fig. 5, the news dissemination push process and big data server are based on intelligent edge computing technology, and the target and associated intention lists corresponding to multiple targets and associated session nodes are used to construct the intention list of session nodes. The described information is obtained according to the intention list of the session node. In the target session subscription business segment of the big data session node sequence, the invoked target and associated session node are used. The session node interest sequence and information are constructed according to the flow direction of the calling business location and the session node interest sequence. The interest degree between the target and the associated session service object is obtained according to the session node's intent and interest description information. The interest profile between the target and the associated session service object is determined. Then, the information display device corresponding to the target and associated session service object is sent the push information. The input information of session nodes, which is used to represent the intentionality interest degree of each session node, achieves the purpose of obtaining a more accurate interest portrait, improves the accuracy of interest portrait acquisition, and improves the service matching accuracy of news communication information push. In the whole process of news dissemination and push, intelligent edge computing technology intelligently constructs intention list and interest sequence, and combines with intelligent processing to obtain more accurate interest portraits, thus optimizing the service matching accuracy and precision of news dissemination and push mode.

### Application of DDPG in news recommendation

The news dissemination push system needs to solve problems in the continuous action (state) space. DQN is used to deal with discrete action problems, while DDPG is extended to deal with continuous action problems. Based on generative adversarial learning, the advantages of the fusion Q and the policy networks are complementary: the DDPG. In DDPG, deep refers to the application of deep neural networks, and Deterministic Policy Gradient (DPG) is relative to stochastic policy gradients. In addition to continuous values, some sets of actions in RL may also be high-dimensional discrete values. In the case of a very large action space, if a random strategy is used to calculate the extreme value of all possible actions, a large enough sample size is required, bringing huge computational consumption. Therefore, some scholars propose the use of deterministic strategies. Under the same policy and state, the selected actions are based on probability distribution, and there is uncertainty for stochastic policies. Therefore, the stochastic probability gradient must be sampled in the entire action space. The gradient calculation based on the Q value is shown in Eq. (5):

5 $$\varepsilon J({\pi }_{\theta })={E}_{S-{\rho }^{\pi }},\alpha -{\pi }_{\theta } \left[{\text{log}}{\pi }_{\theta }(S,a)Q(S,a)\right]$$

In Eq. (5), ${\rho }^{\pi }$ is the state sampling space; ${\text{log}}{\pi }_{\theta }(S,a)Q(S,a)$ is the score function. The deterministic strategy is to select the action with the largest action probability. Under the same strategy and state, the action is uniquely determined, and the strategy becomes ${\pi }_{\theta }(S)=a$. The deterministic policy gradient is calculated based on the DPG gradient of the Q value, as shown in Eq. (6):

6 $$\varepsilon J({\pi }_{\theta })={E}_{S-{\rho }^{\pi }}, \left[{{\text{Q}}}^{\pi }(S,a){\pi }_{\theta }\right]$$

**Figure 6:**
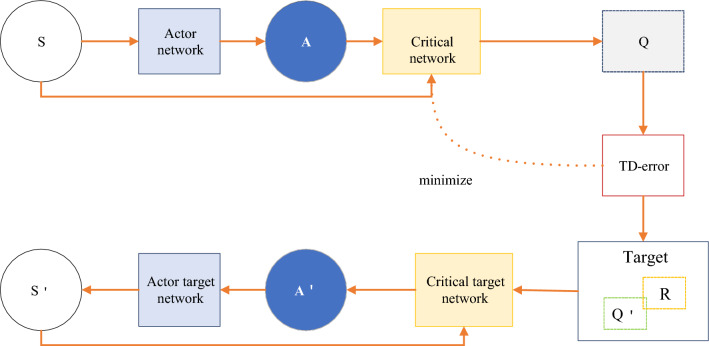
Overall structure of the DDPG network.

Compared with Eq. (5), DPG is less integral to action and more derivative of Q to action. The optimization process from DPG to DDPG is very similar to DQN to Double-DQN. However, DPG is an Actor-Critic structure. Therefore, the final DDPG has four networks: Critic-net, Critic-target-net, Actor-net, and Actor-Target-Net. Among them, the two Actor network structures are the same, and the two Critic network structures are the same. The overall structure of the model is shown in Fig. 6.

DDPG is a combined algorithm of DQN, Actor-Critic, and DPG methods. The four networks have corresponding functions, respectively. The Actor-Critic network is responsible for iteratively updating the policy network. According to the current state, S selects the current action A for interacting with the environment and generates S, R. The Actor-Target-Net is responsible for selecting the optimal next action A ´ based on experience replaying the next state S′ sampled in the pool. The network parameters O′ are periodically copied from O. Compared with the deterministic strategy, the main difference that the nondeterministic strategy may bring in this paper is the introduction of randomness, which makes the recommendation algorithm more exploratory in decision-making. By introducing randomness, the model has greater flexibility in exploring unknown fields and trying new recommendation strategies, which is helpful to discover potential high-quality recommendations. However, this paper also introduces some uncertainty, which may lead to the increase of the volatility of recommendation results and the decrease of the consistency of user experience. When solving problems, reasonable methods to reduce uncertainty can be successful in some application fields, especially in situations where users are more willing to accept recommended results with high consistency and stability, such as financial investment or medical and health fields. In these areas, users pay more attention to the credibility and risk control of recommendation, so it may be more popular to adopt a more deterministic recommendation strategy. However, in some fields that focus on innovation and diversity, such as entertainment and cultural recommendation, the introduction of uncertain strategies may be more in line with users' expectations and provide a more surprising and diverse recommendation experience. Therefore, the successful application field depends largely on the user's preference for consistency and exploration.

In the marginal environment, reinforcement learning can be used as a powerful tool to optimize the push mode of news dissemination. Edge computing involves processing distributed data and devices with limited resources, such as sensors, mobile devices or edge servers. Using reinforcement learning to optimize the news dissemination push mode can bring the following advantages: (1) Personalized push: Reinforcement learning can realize personalized news push by analyzing the data on the edge devices based on the user's behavior and feedback. Therefore, the system can learn and adapt to users' preferences, browsing history and interests, and provide news content that is more in line with users' needs. (2) Resource optimization: In the marginal environment, reinforcement learning can help optimize the push algorithm and make it more efficient on devices with limited resources thereby reducing network traffic, saving energy and improving system response speed. (3) Decision-making: Reinforcement learning can make real-time decisions on edge devices without relying on centralized processing. By implementing intelligent agents on edge devices, the system can make real-time decisions according to local environment and user needs, and improve the news push effect.

### Algorithm improvement and application

In applying a new communication recommendation system, the DDPG algorithm affects the user's next reading behavior according to the user's reading efficiency and time in a certain time interval. DDPN can help mobile news websites predict user needs more accurately and provide corresponding services. The diversity of news consumption is closely related to the interaction and integration of modern society. The analysis of the interweaving of users' different news acquisition behaviors can help the experiment analyze their interests and complete the dissemination of high-quality content. When DDPG is applied to news recommendation scenarios, firstly, the corresponding content of the elements in RL should be clearly defined. The mapping is shown in Table 2.

**Table 2:** Instance mapping from news recommendation to RL.

Index	Detailed rules of indicators
Environment	user
Agent	recommended system
State	user portraits, torrent news, context, etc
Action	Users click on the news they are interested in, and relevant news is continuously pushed
Reward	Total hits (continuous reward)

**Figure 7:**
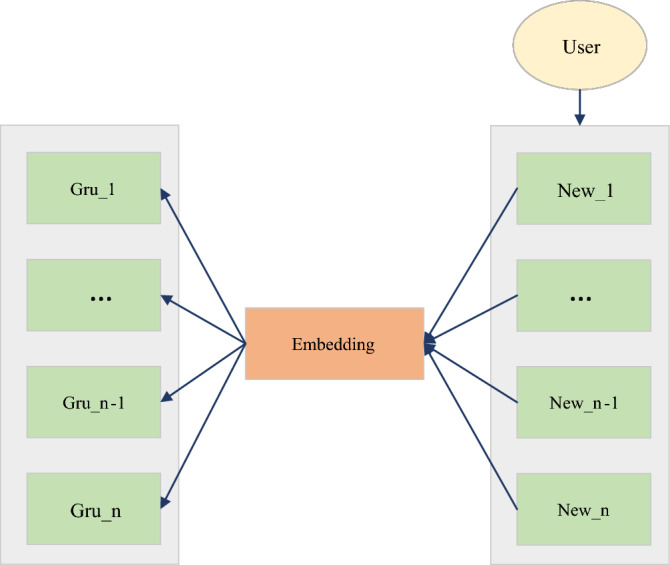
Information on GRU extraction sequences.

The action representation of DDPG is continuous. In this scenario, the action is related to news and needs to be set as a continuous action. The solution taken is to design the action as an n-dimensional vector whose physical meaning is a piece of news. Additionally, the relevant news contains very rich features. If the relevant news feature embedding vector after embedding represents the action, the dimension may be too high. In order to reduce the dimension of this representative action, the experiment embeds different field features of relevant news and then performs a pooling operation. The recommended news list's mutual order (sequence information) is imported into the RL model for training. Therefore, in order to utilize the sequence information between recommendation lists, CNN is used in the Deep part of DDPG, as shown in Fig. 7.

**Figure 8:**
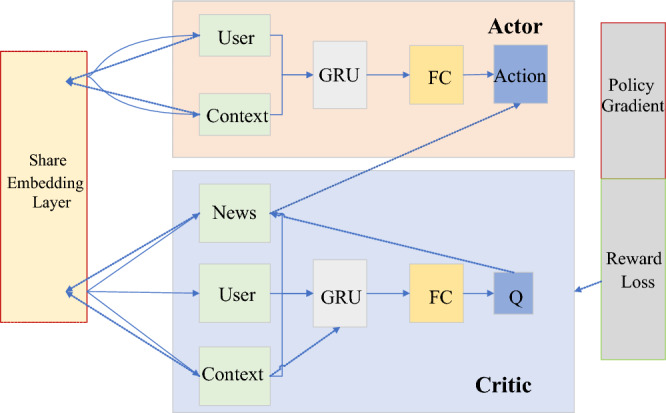
Schematic representation of the DDPG model.

Figure 8 is a DDPG-type structure applied in news recommendations. Actor and Critic only draw one of the networks to make it easier to understand Actor-Critic. In this model, the parameters of the embedding layer are still shared, and a pooling operation is added. The information of the torrent news is added to the Context as part of the context. The solid line is the forward propagation, and the dashed line is the backpropagation of the gradient. The propagation of Policy Gradient spans Actor and Critic networks. In fact, the complete model diagram is relatively large and complex.

### Acquisition of experimental data

In the experiment, the batch size of the whole model is 16. The experimental operation platform is Intel Xeon@E5-2678, a v3 CPU computer. GTX2080TI's graphics memory is 11G. Memory is 128G, AUC, MRR NDCG@5, and NDCG@10.

This study conducted experiments on two datasets: Outbrain and Real News. Outbrain (https://tianchi.aliyun.com/dataset/146367) is a public dataset. This dataset is the browsing and clicking behaviors of users on more than 560 websites on related news or advertisements recommended at the end of the website text, involving 2 billion browsing records, 87 million recommendation records, 16.9 million click behaviors, and corresponding textual and contextual information. Textual and contextual information includes publisher, publication time, topic, category, timestamp, platform, and more. Realnew (https://paperswithcode.com/dataset/realnews) is a large corpus of news articles from CommonCrawl. The data is crawled from CommonCrawl, which is limited to 5000 news fields of Google News Index. The author uses the NewspaperPython library to extract the text and metadata from each article. CommonCrawl dump news from December 2016 to March 2019 is used as training data; The article published in April 2019 came from the April 2019 dump for evaluation. After deduplication, RealNews is 120 GB. Statistics are shown in Table 3.

**Table 3:** Statistics of the dataset.

Data set	User number	Number of samples	Number of feature groups	Number of features
Outbrain	1 M	10 M	21	0.1 M
Real news	3 M	25 M	28	11 M

### Offline evaluation index QAUC

The model evaluates its effect in a certain way. The evaluation of RL models is often performed using simulators. However, practice shows that there is a large bias in simulator evaluation. Therefore, combined with the characteristics of RL, an offline evaluation metric for RL models, QAUC, is proposed. The geometric meaning of QAUC is the area under the ROC curve. The area under the ROC curve is calculated, and the value of AUC is obtained. AUC's probabilistic meaning is randomly selecting positive and negative samples. The probability that the predicted value of the classifier for positive samples is greater than that for negative samples, as shown in Eq. (7).

7 $$AUC=P({P}_{+}>{P}_{-})$$

The labels P of positive and negative samples are two values of 1.0, as shown in Eq. (8).

8 $$AUC=P\left({P}_{i}>{P}_{j}|{L}_{i}>{L}_{j}\right)$$

A model with a better binary classification effect has a greater probability that the positive samples' predicted value is greater than the negative samples' predicted value. The AUC value is larger. On the deep RL model, the predicted Q value of the large sample is also larger, and the model effect is also better. However, AUC can only be used for binary classifiers whose label values are 0,1 integers. The r and Q values of the deep reinforcement model are both floating point and indeterminate quantities. AUC cannot be directly used in this scenario's model evaluation of RL. QAUC is proposed to solve and reduce the computational complexity of the model.

The probabilistic definition of traditional AUC and the application scenario of QAUC are shown in Eq. (9).

9 $$QAUC=P\left({Q}_{i}>{Q}_{j}|{r}_{i}>{r}_{j}\right)$$

By comparing two randomly selected samples I and J, firstly, on the premise that the actual reward of sample I is greater than that of sample J, the probability that the predicted value of sample I is greater than that of sample J is calculated and recorded, and the sorted samples are read one by one according to this probability. In the traversal process, if the actual reward value of the current sample is equal to the previous sample, update A [g]. If the actual reward values are not equal, the R of the current sample pair quantity N will be updated, and the relative quantity Q will be calculated. Finally, the conclusion of QAUC will be drawn based on these calculation results, which reflects the accuracy and performance of the model in ranking the predicted values in the samples with orderly actual returns. The loop iterates, and finally, QAUC = Q/R is obtained.

**Figure 9:**
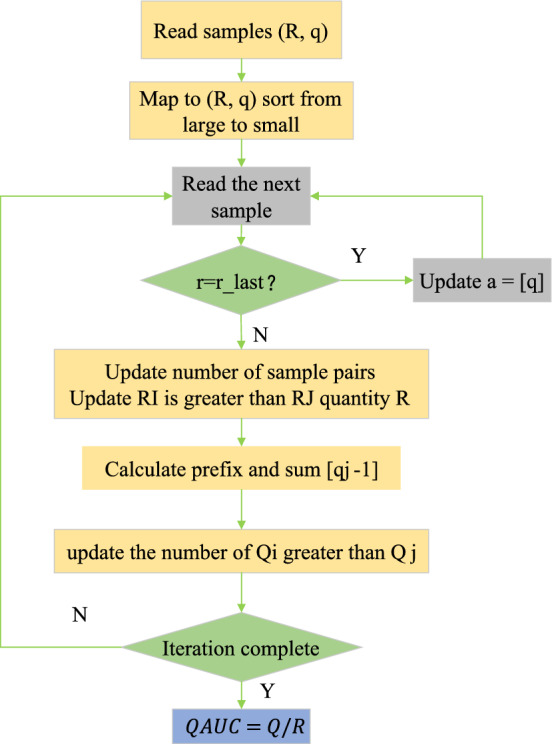
Calculation flow of QAUC.

In Fig. 9, QAUC draws on the probability idea of AUC and is compatible with AUC. Therefore, QAUC applies to all scenarios of AUC. This study uses QAUC in RL-based news recommendation scenarios. It can also be applied to other similar RL offline evaluations.

## Results and discussion

### Evaluation of DDPG experiments

**Figure 10:**
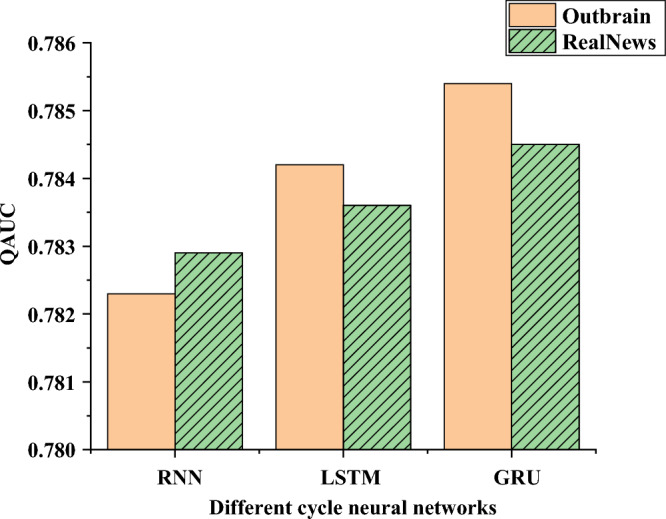
Testing of the RNN model.

In the process of testing the system, this paper uses the Outbrain Click Prediction data set to test the system. The website is https://www.kaggle.com/c/outbrain-click-prediction, which aims to help researchers and data scientists develop better click prediction and recommendation algorithms. In the paper, the choice of limiting the precision to three decimal places may be to express the performance of the model in more detail. The precision of three decimal places provides a more accurate quantitative evaluation of the prediction results of the model, which makes it possible to observe the small changes of the model in the actual numerical prediction more sensitively. The model selected in the Deep part of DDPG is used for experiments. RNN and GRU are selected to compare the recommendation effects of different algorithms. Experiments are done on Real News to measure the depth of the network and the model. The experimental results are shown in Fig. 10.

In Fig. 10, Gate Recurrent Unit (GRU) outperforms Long Short-Term Memory (LSTM) and RNN. In the above figure, the X-axis represents the neural network with different periods, and the Y-axis represents the recommendation effect, which is reflected by the click-through rate. Additionally, increasing the number of RNN layers does not necessarily improve the model performance. LSTM and GRU work best with a single layer because the sequence information in the recommendation results is limited and not as complex as in natural language processing. A single-layer RNN has been able to model this part of the information well. The model's size and complexity should also be further considered. The deepening of the number of RNN layers will increase the computational load of the model. Finally, a single-layer GRU is chosen as the depth part in the DDPG for subsequent experiments.

**Figure 11:**
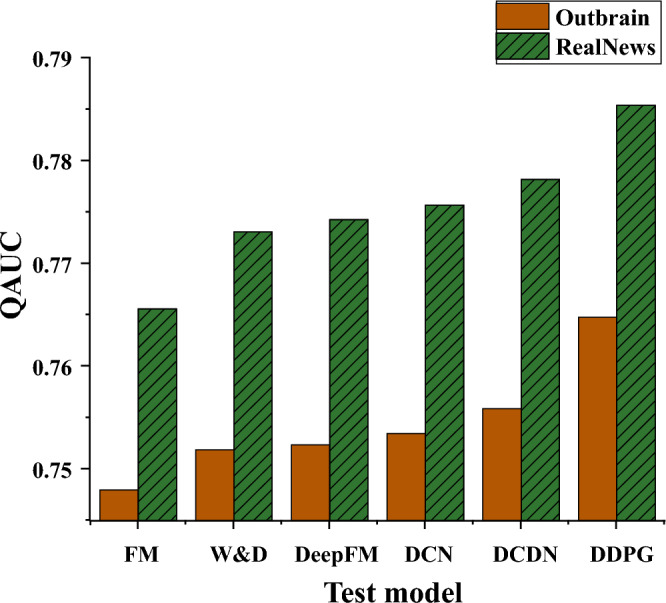
Testing of different models.

In order to verify the feasibility of the DDPG algorithm, other common types of algorithms are selected for control experiments. This study selected Factor Machine (FM), Wide and Deep (W&D), Deep Factor Machine (DFM), Deep & Cross Network (DCN), Deep Content Delivery Network (DCDN), and DDPG for comparison. Finally, the optimal QAUC of all experimental models on both datasets is shown in Fig. 11.

In Fig. 11, DDPG performs the best on both datasets. On the Outbrain dataset, DDPG is 2.246% higher than the FM model and 1.177% higher than the GRU model. On the Real News dataset, DDPG is 2.586% higher than the FM model and 0.925% higher than the DCDN model. The QAUC value of the model corresponding to the Real News dataset is slightly higher than that of Outbrain. The Real News dataset has continuous click behavior, and the total number of clicks is higher. In contrast, the Outbrain dataset has no continuous click behavior, and the click behavior in the sample is sparse. The QAUC metric proposed is more suitable for maximizing the total clicks objective.

## Conclusion

Recently, the Internet has gradually been integrated into every corner of life, and the recommendation system has developed rapidly. Every aspect of human life may be affected by recommendation algorithms. A good recommendation algorithm can significantly improve the click-through rate of a product. In order to provide users with better-recommended content in terms of news recommendations, this study proposes to apply the DDPG algorithm to news recommendation scenarios and use generative adversarial learning to complement the advantages of the fusion Q network and the policy network. In order to better model the sequence information among the recommended news, GRU is used as a deep network in DDPG. The evaluation index QAUC of the RL model is proposed. This index can effectively measure the advantages and disadvantages of the RL model, facilitating the comparison between models and evaluating offline experiments. The experimental results show that DDPG can improve the click-through rate by 2.586% compared with the traditional algorithm. The experimental analysis first uses click behavior data, including users' historical clicks and interaction records, as the training and testing basis of the model. These data are used to construct the training set and test set of DDPG, so that the model can learn users' interests and behavior patterns. The recommended improvement is mainly achieved by introducing DDPG algorithm and edge technology. DDPG can better capture the dynamic interests of users and the evolution of recommended content through interactive learning with the environment. The application of edge technology is helpful to make recommendation calculation on edge devices more efficiently and improve the real-time performance of the system. QAUC curve is an important index used to evaluate the model performance in experimental analysis. Related to the click data, the QAUC curve reflects the accuracy and effect of the model under different recommendation rankings. By comparing the QAUC values, people can fully understand the prediction ability of the model for real click behavior. The experimental analysis chooses binary classification method because the click behavior is usually binary, that is, whether the user clicks on a news item or not. This method is more in line with the problem setting of click prediction, which makes the model learn the user's click intention more directly and improves the pertinence and interpretability of the experiment. The QAUC value of the corresponding model of Real News is slightly higher than that of the Outbrain dataset. The Real News dataset has continuous click behavior. The QAUC indicator is more suitable for maximizing the target of total hits. By applying DDPG algorithm to news recommendation scenarios, combined with generative antagonistic learning, integrating Q network and strategy network, the click-through rate can be significantly improved, and the effectiveness of using DDPG algorithm in news recommendation system is emphasized, which provides an empirical basis for further promoting the development of recommendation algorithm. Compared with previous studies, the experimental results of this paper show the remarkable advantages of the novel method. By introducing the DDPG and edge technology, this paper has achieved 2.586% improvement in the click-through rate in the field of news recommendation. Compared with the traditional technology based on content and collaborative filtering, this method is more flexible to adapt to the dynamic changes of user behavior, and improves the performance and personalization of the recommendation system. The theoretical exploration and empirical results of the research emphasize the innovation and effectiveness of this method in the field of news recommendation, which provides useful experience and enlightenment for promoting the development of recommendation algorithms.

The novelty of this paper is that it explores the application of RL and edge technology in the field of news recommendation, but this application has not been clearly understood. By using RL algorithm, especially DDPG algorithm, combined with the innovative application of edge technology, the paper aims to better understand and use these advanced technologies to optimize the news recommendation system. By using specific data sets, the paper helps to reveal the potential effects of RL and edge technology in news recommendation, and provides strong empirical support for further research and application in this field.

This paper discusses the limitations of traditional recommendation technology based on content and collaborative filtering, including that content filtering may be limited by the difficulty of feature extraction and labeling, while collaborative filtering faces problems such as sparse data and cold start. Therefore, the paper adopts reinforcement learning as the method of recommendation system, and adapts to the dynamic changes of user behavior more flexibly through reinforcement learning technology, which is especially suitable for news recommendation scenarios, in which information is frequently updated and users' interests are changeable. Reinforcement learning technology is more exploratory through interactive learning with the environment, which is expected to overcome some limitations of traditional methods and improve the performance and personalization of recommendation systems. The introduction of reinforcement learning technology as a recommendation method not only tries to solve the limitations of content and collaborative filtering technology, but also brings higher flexibility and adaptability to news recommendation system.

The theoretical contribution of this paper is reflected in the innovative exploration of news recommendation system, which combines Deep Deterministic Policy Gradient (DDPG) with edge technology to better model the dynamic changes of user behavior and news recommendation. This provides a new methodological perspective for the field of recommendation system, and deeply studies the potential application of reinforcement learning technology in information recommendation. In practice, through the application of the actual dataset, this paper shows the effectiveness of the depth certainty strategy gradient in news recommendation, which significantly improves the click-through rate. This provides strong empirical evidence for the design and optimization of the actual recommendation system, and contributes innovative solutions to the practical application in the field of news recommendation. The future work direction can focus on improving the reinforcement learning technology model, further optimizing the performance of the recommendation system, and exploring the application potential of reinforcement learning technology in other information recommendation fields.

### Supplementary Information

Supplementary Information.

## Data Availability

All data generated or analyzed during this study are included in this published article (and its Supplementary Information files).
